# New, late-onset relapsing-remitting multiple sclerosis in a woman in her 60s after SARS-CoV-2 vaccination

**DOI:** 10.1016/j.clinsp.2024.100556

**Published:** 2024-12-13

**Authors:** Carla Alexandra Scorza, Fulvio Alexandre Scorza, Josef Finsterer

**Affiliations:** a Universidade Federal de São Paulo (UNIFESP/EPM), São Paulo, SP, Brazil; b Neurology Dpt., Neurology & Neurophysiology Center, Vienna, Austria

## Correspondence

There are numerous indications that SARS-CoV-2 Vaccinations (SC2Vs) are not free of side effects and can trigger various neurological and non-neurological diseases.^1^ Most commonly, SC2Vs trigger immunological diseases, most likely due to dysregulation of the immune response to the vaccine.^2^ One of the immunological neurological diseases that can be triggered by SC2Vs is multiple sclerosis.^3^ Several patients have been reported to develop multiple sclerosis after SC2V,^3^, ^4^, ^5^ but no patient over 60 years with new-onset multiple sclerosis after SARS-CoV-2 vaccination has been found in the literature.

The patient is a 67-year-old Caucasian woman with well-controlled arterial hypertension and right cataract surgery who suffered a mild SARS-CoV-2 Infection (SC2I) complicated by headache, retinal hemorrhage in the right eye, partial external ophthalmoparesis of the right globe and double vision in 2020. Cerebral imaging had not been performed at that time. With the exception of the double vision and strabismus, all other symptoms and signs resolved completely. In May 2022, she received her first dose of an mRNA-based anti-SARS-CoV-2 vaccine. Four weeks later, she received the second dose from the same manufacturer. She had no acute complications from these vaccinations. However, three months after the second vaccination, the patient complained of general fatigue, easy fatigability, blurred vision, gait disturbances, and distal hypoesthesia of the lower limbs. There was no clinically manifest infection or fever between the second vaccination and the onset of these symptoms. She did not report any previous visual disturbances, sensory deficits, or urinary problems.

When these complaints were investigated in October 2022, mild left-sided hemiparesis and sensory disturbances in the lower limbs were detected. Cerebral MRI showed a right frontal T2 hyperintense lesion that increased slightly with contrast. CSF examination revealed positive Oligoclonal Bands (OCBs) but was otherwise normal. Cerebral Isolated Syndrome (CIS) was diagnosed. As symptoms progressed, she underwent a second examination in March 2023, which revealed a pale papilla, convergence weakness, strabismus, left-sided hemiparesis M3, increased tendon reflexes in the lower limbs, positive pyramidal signs in the lower limbs, sensory disturbances in the distal lower limbs and an uncertain Romberg test. Cerebral MRI showed enlargement of the previously described lesion in the right frontal lobe and a new lesion in the right parietal lobe. As the patient fulfilled Mcdonald's criteria (spatial and temporal spread), multiple sclerosis was diagnosed and treatment with dimethyl fumaric acid ester 240mg once daily was started.

**Fig. 1:**
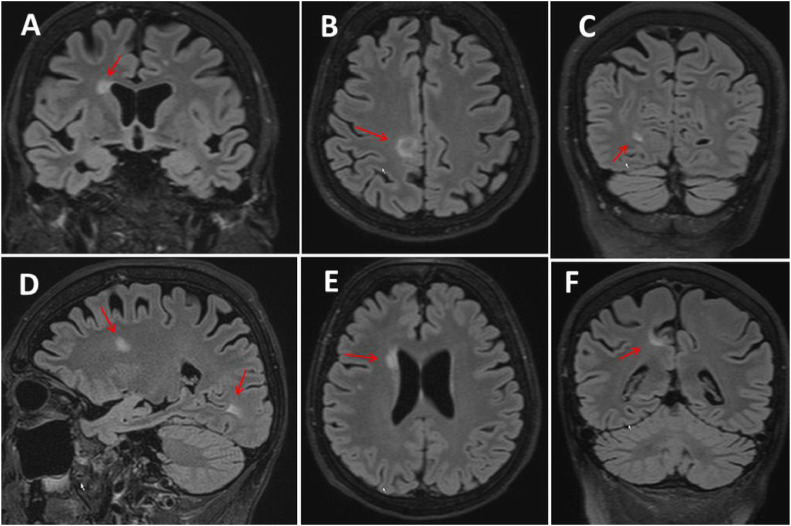
Cerebral MRI from January 2024 showing multiple T2 hyperintense lesions in the right hemisphere, especially in the right frontal lobe perpendicularly (panels A, D, E), in the parietal lobe (panels B, F), and the right occipital lobe (panels C, D).

Under this treatment, her symptoms regressed, and in March 2024, the clinical neurological examination revealed only short stature, myopia, a non-circular right pupil, a deviation of the right bulb to the midline, a slight paralysis of the right abducens, double vision when looking straight ahead and to the right, lack of Achilles tendon reflexes, left hemihypalgesia, pallhypaesthesia in the distal lower limbs, unsteadiness in the Romberg test and a tendency to fall in the Unterberger test and the line walk. The other reflexes were lively, in favor of the left side. The Expanded Disability Score (EDDS) was 1.5. Cerebral MRI showed typical multiple sclerosis plaques in the right parietal region, a second subcortical high parietal, and a third in the right occipital lobe (Fig. 1). None of them enhanced. Aquaporin-4 antibodies, Myelin Oligodenderocyte Glycoprotein (MOG) antibodies and Neuromyelitis Optica (NMO) antibodies were negative. The visual evoked potentials were normal. She was recommended to increase the fumaric acid but preferred to maintain the previous dosage.

The presented patient is interesting because of late-onset multiple sclerosis after SC2V with an RNA-based vaccine. Whether the cerebral lesion already developed after SC2I in 2020 or only after the first or second SC2V remains speculative, as the earliest imaging examinations were only performed in October 2022.

Multiple sclerosis that begins at the age of 67 is unusual and requires clarification as to whether the diagnosis is really correct or not. The fact that the number of cerebral plaques increased over a period of two years, that she fulfilled Mcdonald's criteria for the diagnosis of multiple sclerosis, and that the OCBs were positive speaks in favor of multiple sclerosis in the index patient. The fact that the visual evoked potentials were normal, that the CSF/serum IgG ratio was normal, and that the age at onset of the disease was unusual for multiple sclerosis argues against multiple sclerosis. A causal relationship between SC2V and multiple sclerosis is supported by the fact that there was no other infection or vaccination that could be held responsible, that a causal relationship has been previously reported,^3^, ^4^, ^5^ that SC2V can trigger or exacerbate other immunological diseases^6^^,^^7^ and that the patient had no symptoms of multiple sclerosis prior to SC2V. Whether SC2I and SC2V reinforce each other as triggering factors for multiple sclerosis remains speculative, but a cumulative effect of SC2V is conceivable. Whether SC2V additionally accelerated immunosenescence, as previously reported in other patients,^8^ and promoted the development of immunological side effects of SC2V remains speculative.

In conclusion, this case shows that SC2V and/or SC2I either together or alone can trigger the development of multiple sclerosis, even in a patient over sixty years of age. Further studies are needed to assess whether there really is a causal relationship between SC2V and new-onset multiple sclerosis.

## Ethical approval

Not applicable.

## Consent to participation

Not applicable.

## Consent for publication

Not applicable.

## Availability of data and material

All data are available from the corresponding author.

## Authors’ contributions

JF was responsible for the design and conception, discussed available data with coauthors, wrote the first draft, and gave final approval. SM: contributed to literature search, discussion, correction, and final approval.

## Funding

None received.

## Conflicts of interest

The authors declare that the research was conducted in the absence of any commercial or financial relationships that could be construed as a potential conflict of interest.
